# Racial and Educational Isolation are Associated with Worse Outcomes in Congenital Heart Disease

**DOI:** 10.21203/rs.3.rs-5290293/v1

**Published:** 2024-10-30

**Authors:** Meredith Sooy-Mossey, Neeta J. Sethi, Hillary Mulder, Karen E. Chiswell, Timothy M. Hoffman, Robert J. Hartman, Michael J. Walsh, Karl Welke, Joseph A. Paolillo, Lauren A. Sarno, Charlie J. Sang, Alfred D’Ottavio, Claire Osgood, Mercedes A. Bravo, Marie Lynn Miranda, Jennifer S. Li

**Affiliations:** Duke University School of Medicine; Duke University School of Medicine; Duke Clinical Research Institute; Duke Clinical Research Institute; University of North Carolina at Chapel Hill Medical Center; University of North Carolina at Chapel Hill Medical Center; Wake Forest University School of Medicine; Atrium Health Levine Children’s Congenital Heart Center; Atrium Health Levine Children’s Congenital Heart Center; Brody School of Medicine at East Carolina University; Brody School of Medicine at East Carolina University; Duke Clinical Research Institute; University of Illinois Chicago; Duke University; University of Illinois Chicago; Duke University School of Medicine

**Keywords:** Complex Congenital Heart Disease, Health Disparities, Race, Racial Isolation, Educational Isolation

## Abstract

**Background:**

Sociodemographic factors influence outcomes in children with congenital heart disease (CHD). We predict an association between measures of social isolation and outcomes in infants with complex CHD. These measures, racial (RI) and educational (EI) isolation range from 0 to 1, with 0 being no isolation and 1 being fully isolated within a specific population.

**Methods:**

We identified patients less than 1-year-old undergoing CHD surgery in North Carolina from 2008–2013. We used negative binomial and logistic regression models to assess the case-mix adjusted associations between RI and EI and length of stay, complications, mortality, and resource utilization. We quantified the association of race with these indices and outcomes.

**Results:**

We included 1,217 infants undergoing CHD surgery. Black infants had increased LOS (p < 0.001), other complications (p = 0.03), and death (p = 0.02). RI up to 0.3 was associated with decreased outpatient encounters (p < 0.001). RI was associated with increased inpatient encounters RI up to 0.3 (p < 0.001) but decreased for RI beyond 0.3 (p = 0.01). There was an association with increased risk of one or more emergency department visits (p = 0.001) at higher levels EI. Race and RI showed a cumulative effect with children with Black race and greater than median RI having increased LOS (p < 0.001) and fewer outpatient encounters (p = 0.02).

**Conclusions:**

RI, EI, and Black race are associated with poorer outcomes. Children with Black race and greater than median levels of RI are at the highest risk of poor outcomes. These differences may be caused by differential access to resources or community support.

## Introduction

Congenital heart disease (CHD) occurs in approximately 1% of births, representing the most common congenital disorder in newborns with many of these diagnoses having significant morbidity and mortality.^1^ Like other health conditions, patient race, ethnicity, and socioeconomic status (SES) play a role in outcomes in children with CHD. Black children have been shown to have increased mortality compared to White children with CHD across a variety of types of CHD lesions.^2–5^ Patients with Hispanic ethnicity have increased in-hospital mortality compared to Non-Hispanic patients.^6–9^ Children with public insurance have increased mortality compared to those with private insurance.^2,6,7,9^ Maternal education level has shown an unclear association with mortality in children with CHD, with some studies showing that lower maternal education is associated with increased mortality.^3,4,10^ In addition to individual level factors, there is evidence that neighborhood level factors play a role in outcomes in children with CHD.^11,12^ Children from the lowest income neighborhoods have an increased odds of mortality and longer length of stay (LOS) when undergoing CHD surgery.^11^

A recent statement by the American Heart Association identified the importance of individuals’ neighborhoods in shaping their “environmental exposures, access to resources, and opportunities” that affect their health.^13^ There are many aspects of neighborhood composition postulated to affect patient outcomes. Williams and Collins (2001) suggest that racial isolation is a leading component of health disparities with racial segregation leading to disparate economic and educational opportunities, which persists in today’s society due to structural racism.^14,15^ Residential racial segregation is a multifaceted concept characterized by five components including evenness, isolation, concentration, centralization, and clustering.^16^ Racial isolation (RI) can be measured at varying geographic scales and fundamentally aims to describe the degree to which individuals are surrounded by other individuals within a specific racial group within their neighborhood environment. This measure which looks at an individual’s neighborhood surroundings offers a way to characterize an individual’s living environment, which includes levels of poverty, levels of unemployment, housing quality, access to healthy foods, and access to transportation, as these measures have been shown to have an association with racial segregation^17^. Increased rates of RI are associated with increased risks of preterm birth and low birth weight and may similarly play a role in outcomes in children with CHD. ^18–21^ A similar measure, educational isolation (EI), describes the degree to which individuals are isolated by educational attainment. As above, maternal education level has shown mixed results in association with mortality outcomes in children with CHD.^2,4,22–24^ There are sparse data utilizing EI and its association with health outcomes.

**Figure 1:**
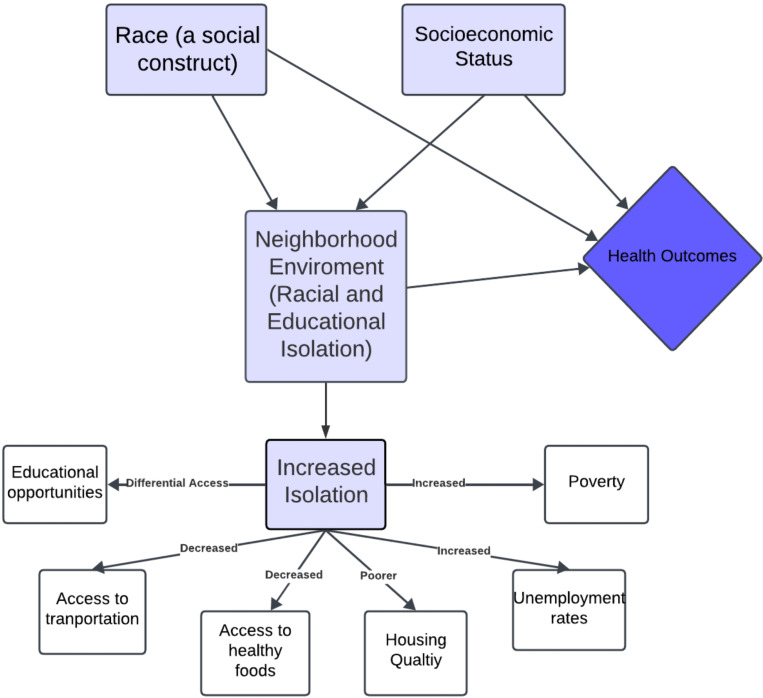
Racial and educational isolation conceptual model

RI and EI represent the degree to which individuals are isolated by racial and educational composition within a defined population.. These indices are created by matching an individual to their census block group a division of a census tract which typically includes between 600 to 3,000 individuals. Block group level scores are calculated by accounting for the population composition in the index block group where an individual resides as compared to adjacent blocks to calculate a RI and EI value. These values range from 0 to 1 and represent the degree of isolation of the index block where the patient resides compared to the surrounding blocks. A RI of 0 indicates that the neighborhood environment (defined as the index block group and adjacent block groups) is 100% non-Non-Hispanic Black (all races other than Non-Hispanic Black), and 1 indicates that the neighborhood environment was 100% Non-Hispanic Black (NHB).^21^ For EI, a score of 0 indicates an environment composed entirely of college graduates and a score of 1 indicates an environment composed entirely of non-college graduates. RI and EI do not reflect an individual’s racial or educational status, rather the racial or educational composition of the census block in which they reside compared to its surrounding blocks. These measures provides a spatial measure of segregation and may be closely related to an individual’s health ^17,25^ Individual patient factors (race and socioeconomic status) and neighborhood level factors (RI and EI) form a complex environment which ultimately affects patients’ health outcomes (Fig. 1).

We aimed to use RI and EI to determine whether disparities exist in outcomes in infants with CHD requiring surgical intervention in the first year of life. We predicted that patients with increased levels of RI and EI would have worse outcomes when compared to patients with lower RI and EI.

## Methods

### Data source and population

Data for this study were obtained through the North Carolina Congenital Heart Defects (NCCHD) Surveillance Network. This database is composed of 50,429 patients with CHD, as identified by International Classification of Diseases, Ninth Revision (ICD-9), code recorded in the electronic medical record for a healthcare encounter between 2008 and 2013 at one of five comprehensive or affiliated satellite CHD centers (Duke, University of North Carolina, East Carolina University, Wake Forest, and Atrium Health). For the analysis reported here, infants undergoing congenital heart surgery between 2008–2012 identified by procedure code at less than one year of age were evaluated. Follow-up data were collected through 2013. Electronic health records were linked to state birth and death certificate records and to the Society of Thoracic Surgeons Congenital Heart Database. Patients undergoing patent ductus arteriosus ligation and heart transplantation were excluded. Patient RI was determined using 2010 census block group level data with address at the time of index hospitalization. Type of CHD was divided into severe, shunt, valve, or other based on ICD-9 code (supplementary Table S5). The groupings of severe, shunt, and valve were predetermined by the CDC and Prevention Congenital Heart Defect Surveillance Network using a previously described method that categorizes CHD diagnostic codes into mutually exclusive hierarchical groups integrating both hemodynamic severity and basic anatomical characteristics.^26,27^ This study was approved by the Duke Health Institutional Review Board and funded by the CDC grant NU50DD004933-01.

### Primary Exposures

Exposures of interest included patient race, RI, and EI. Race was collected from electronic health records and recorded as White, Black, or Other. Other race included the following races: Asian, American Indian or Native Alaskan, and Native Hawaiian or other Pacific Islander. Given small number of patients in each group included in the other category in the study population, the decision was made to combine these into one category. Ethnicity was collected from the electronic health record as Hispanic or non-Hispanic and not included with race data. RI and EI were determined by matching a patient to their census block group and calculating RI and EI using previously describe methodology.^17^ The reference group for RI was individuals who are a race other than NHB (non-NHB). The reference group for EI was college education.

### Primary Outcomes

Outcomes assessed during index encounter include LOS (days) and surgical complications (major and other). Outcomes assessed during 1-year post discharge include days alive and out of hospital, readmission, death, number of outpatient encounters, having at least one emergency department (ED) visit, and having at least one unplanned inpatient admission at participating institutions. LOS of index encounter was calculated using initial admission date to discharge date of first encounter in which patients had a qualifying ICD-9 or CPT code. See supplementary table S4 for full list of ICD-9and CPT codes. Surgical complications were identified by ICD-9 codes and chosen based on cardiac specific complications from the Society of Thoracic Surgeons Congenital Heart Surgery Database.^28^ See supplementary table S6 for full list of complications and ICD-9 codes. Days alive and out of hospital were calculated by subtracting days admitted from the number of days in one year. Death included any type of mortality during the study period. Number of outpatient encounters was calculated as the total number of outpatient cardiology visits the patient had during the study period. Having at least one ED visit and unplanned inpatient admission were dichotomized into no ED visits/ admissions or one or more visits/ admissions.

### Statistical analysis

Demographics of the cohort, including age, sex, race (White, Black or African American, Other, unknown), ethnicity (Hispanic or Non-Hispanic), and insurance type (Medicaid, private, self-pay, or other), and outcomes were summarized by RI and EI tertiles and by race. Tertile 1 represented those with the least isolation and tertile 3 represented those infants with the highest degree of isolation. For continuous variables, mean and standard deviation are presented. For categorical variables, counts and percentages are presented.

The association between RI and EI with outcomes of interest was assessed. For analyses of complications, readmission, binary resource utilization and death, a logistic regression model was used. For analyses of LOS, days alive and out of hospital and number of outpatient encounters, a negative binomial model was used. The relationship between RI, EI, and outcomes was evaluated for non-linearity using natural cubic splines. Non-linear relationships were modeled using two linear piece-wise splines in the final models. Adjusted analyses were performed utilizing the following variables: age (days), sex, race, ethnicity, disease severity, and preterm birth (gestational age < 37 weeks). Death was not modeled in the adjusted setting due to small event counts. Rate ratios and odds ratios with 95% confidence intervals (CI) were presented for every 0.10 unit increase in the evaluated index.

Additional models were fit to evaluate the potential for an interaction between race and RI. For the purpose of describing the cumulative effect of race and RI on outcomes, RI was dichotomized using the median value, and patients with Black or White race were grouped into four classes (Black patients with RI < median, Black patients with RI ≥ median, White patients with RI < median, and White patients with RI ≥ median). All models included both RI and race. Rate ratios and odds ratios were presented with Black or White race and RI < median as the reference categories. Adjustment variables were the same as those previously specified.

## Results

### Characteristics of Infants Overall and by Racial Isolation and Educational Isolation

Of the 50,429 unique patients in the database, 6,816 had a procedure code of interest. An index procedure occurred in infants less than one-year-old in 1,558 infants and 1,307 had the procedure performed between 2008–2012. Of these, 1,217 infants had available racial and education isolation data and were included in the final cohort (Supplementary Figure S1).

The median age of the infants in the cohort was 17 days (Table 1). The cohort had 43.3% female infants. The majority of infants had severe CHD (63.4%) defined by ICD-9 code. There was a diverse racial make-up with 22.2% Black, 57.4% White, 13.6% Hispanic 3.2% other, and 17.2% unknown. Many infants had Medicaid (34.3%) followed by private insurance (24.1%). Few patients were self-pay (0.5%) or other insurance (2.0%); however, there was a high rate of missing data for insurance status. The mean RI index was 0.22 (SD 0.17). The mean EI index was 0.74 (SD 0.15).

The median LOS across the cohort was 16 days during the index hospitalization (Table 1). The rate of major complications was 18.3%, however there was a higher rate of other complications (41.1%). There was a 18.2% rate of unplanned readmission one-year post discharge. The median number of days alive and out of hospital during the year post-discharge was 361 days. One-year mortality was 6.8%. In terms of resource utilization in the year post-discharge, mean number of outpatient encounters was 5.6. Most infants had at least one unplanned inpatient encounter (54.5%). There were modest rates of at least one ED encounter (19.8%).

### Characteristics and Outcomes by Patient Race

The mean ages and sex distribution were similar across races. There was a higher percentage of Medicaid insurance in Black infants (40.7%) compared to the White (27.0%) and Other race (25.6%) infants. CHD severity was similar across races. RI was higher in Black infants (0.36) compared to White (0.17) and Other race (0.21) infants. EI was higher in Black infants compared to infants of White and Other races (Supplementary Table S1).

Black infants had a longer LOS at index hospitalization (42.1 days) than White infants (27.0 days,) (Supplementary Table 1). Infants of Other races had a mean LOS 35.4 days. The rates of other complications were higher in Black infants (47.9%) and other race infants (51.3%) than in White infants (38.1%). Black infants had the fewest out of hospital days in the first year with a mean of 310.3 days compared to White (338.2 days) or Other race infants (350.5 days,). Black infants had an increased rate of 1-year death (12.1%) compared to White (5.1%) and Other race (2.6%) infants. Black infants had a lower mean number of outpatient encounters (mean 4.5) compared to White (mean 5.9) or Other (mean 5.3) race infants.

In unadjusted analysis, Black patients compared to White patients had an increased median LOS (RR 1.49, 95% CI 1.25–1.78) and increased odds of other complications (OR 1.49, 95% CI 1.07–2.08) and death (OR 2.18, 95% CI 1.21–3.93) (Table 2). After adjusting for racial isolation, age, sex, ethnicity, disease severity, and preterm birth, LOS remained significant with Black infants having increased LOS (RR 1.63, 95% CI 1.38–1.91) while other complication (OR 0.99, 95% CI 0.66–1.47) and at least one ED encounter (OR 1.51, 95% CI 0.98–2.34) were no longer significant. Death was not modeled in adjusted setting due to small counts.

### Outcomes by Racial Isolation

Infants had similar age, sex, amount with Hispanic ethnicity, and disease severity across RI tertiles (Supplementary Table S2). There was a lower percentage of private insurance in the most isolated tertile (tertile 3) compared to the least isolated tertile (tertile 1). The mean LOS and 1-year morality rate were highest in the most isolated tertile (tertile 3) (36.3 days and 9.0%, respectively). The mean number of outpatient visits was highest in the least isolated tertile (tertile 1) (6.5 in tertile 1 vs 4.7 in tertile 3).

**Figure 2:**
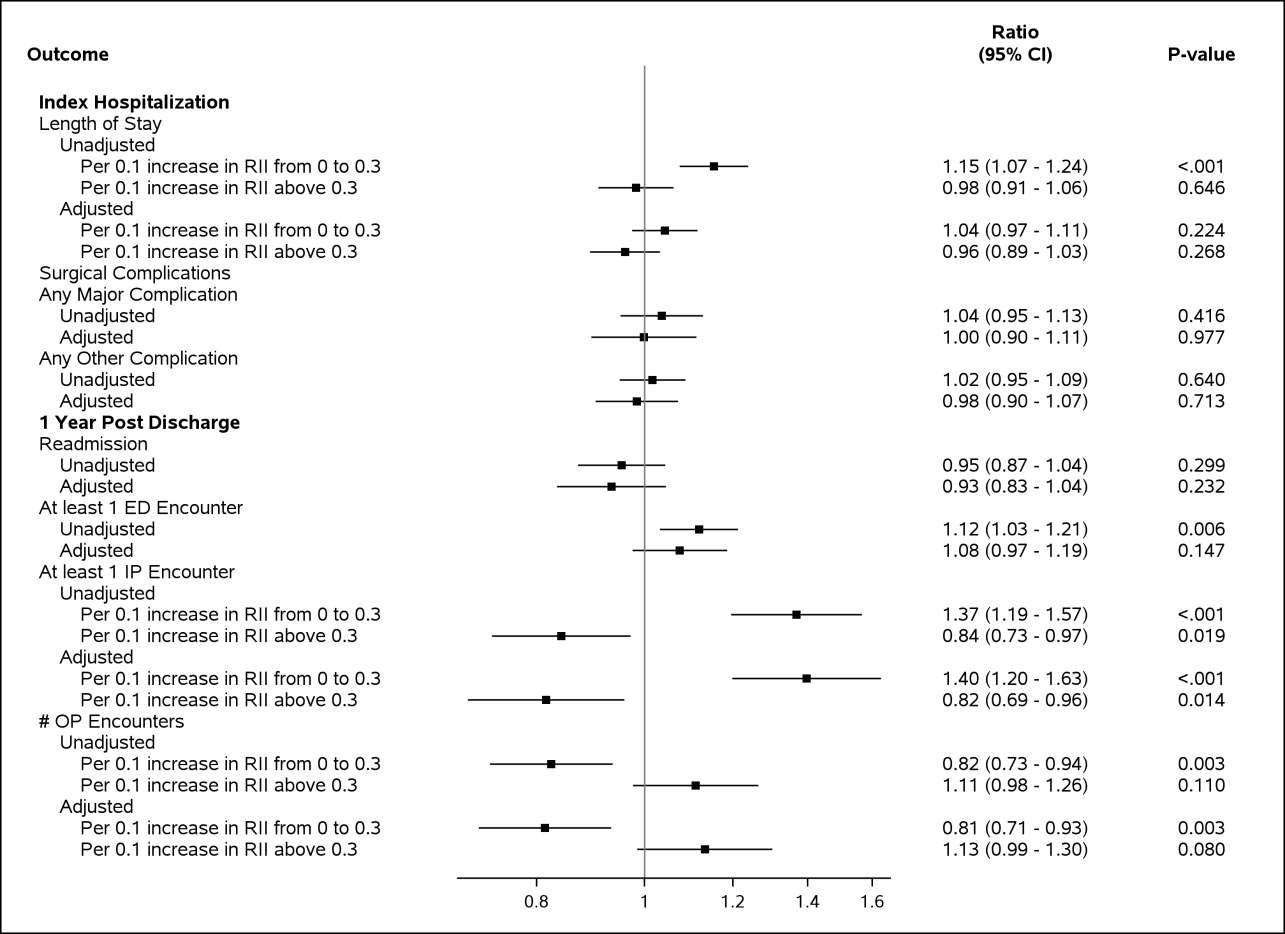
Association of Racial Isolation and Outcomes Length of stay was modeled using negative binomial regression. The estimate presented is the incidence rate ratio. All other outcomes were modeled using logistic regression and the estimate presented is the odds ratio. *1Racial isolation index was non-linearly related to length of stay, OP encounters and IP encounters. Linear splines were created at an inflection of 0.3. *2Adjustment variables include age (days), sex, race, ethnicity,and disease severity SD= Standard deviation, RII= racial isolation index, EII= educational isolation index, OP= outpatient, ED= Emergency department, IP=inpatient

In unadjusted analysis there was an increased LOS (up to RI 0.3), mortality, odds of having at least one ED visit and at least one inpatient encounter (up to RI 0.3) with increasing RI (Fig. 2). There was a decrease in the number of outpatient encounters and odds of having at least one inpatient encounter with increments in RI greater than 0.3. When adjusting for age, sex, race, ethnicity, disease severity and LOS, an increased odds of at least one unplanned inpatient encounter remained significant (OR = 1.39, 95% CI 1.20–1.63 per 0.1 increment in RI). Additionally, there remained decreased number of outpatient encounters at increments in RI up to 0.3 (RR = 0.81, 95% CI 0.71–0.93) and decreased odds of at least one unplanned inpatient encounter for increments above an RI of 0.3 (OR 0.82, 95% CI 0.69–0.96). There was no difference in LOS or number of outpatient encounters for increments in RI above 0.3 or risk of major or other complications.

### Outcomes by Educational Isolation

There was a similar age, sex, amount with Hispanic ethnicity, and preterm birth rate across EI tertiles (Supplementary Table S3). There was a higher percentage of infants with private insurance in the least educationally isolated tertile (tertile 1) and higher percentage of infants with Medicaid insurance in the most isolated tertile (tertile 3). Hospital readmissions and mortality rate were highest in the most isolated tertile (20.3% and 8.6%, respectively). This group also had the lowest number of days alive and out of hospital (321.9 days).

**Figure 3:**
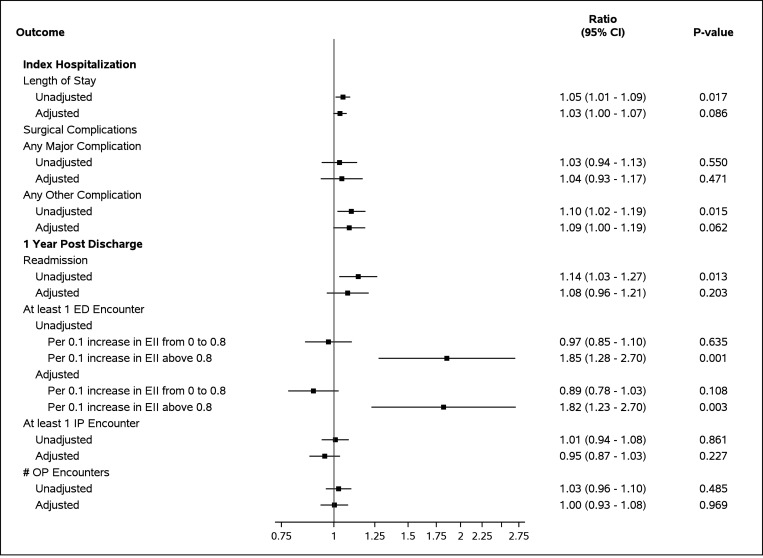
Association of Educational Isolation and Outcomes Length of stay was modeled using negative binomial regression. The estimate presented is the incidence rate ratio. All other outcomes were modeled using logistic regression and the estimate presented is the odds ratio. *1Adjustment variables include age (days), sex, race, ethnicity, and disease severity. *2Educational isolation index was non-linearly related to ED encounters. Linear splines were created at an inflection of 0.8. SD= Standard deviation, RII= racial isolation index, EII= educational isolation index, OP= outpatient, ED= Emergency department, IP=inpatient

In unadjusted analysis there was an increased risk of longer LOS (RR1.05, 95% CI 1.01–1.09), other complications (OR 1.10, 95% CI 1.02–1.19), hospital readmission (OR 1.14, 95% CI 1.03–1.27) and at least one ED visit with increased educational isolation at EI above 0.8 (OR 1.85, 95% CI 1.28–2.10) (Fig. 3). After adjustment for age, sex, race, ethnicity, disease severity, preterm birth and length of index hospitalization, increased risk of at least one ED visit remained significant with increased educational isolation at EI above 0.8 (OR 1.82, 95% CI 1.23–2.70). There was no difference in odds of major surgical complications, mortality, number of outpatient encounters, and having at least one unplanned inpatient encounter.

### Cumulative Association of Race and Racial Isolation with Outcomes

When assessing the interaction between patient race and racial isolation we found little evidence that race modifies the association of RI with outcomes (all p-values but one was not statistically significant, data not included). When we compared patient outcomes based on race and RI, Black infants with greater than or equal to median RI had the highest mean LOS (RR = 1.69, 95% CI 1.42–2.02) relative to White infants with less than median RI, and the highest odds of mortality (OR 3.40, 95% CI 1.84–6.32), and at least one inpatient encounter (OR 1.62, 95% CI 1.16–2.26) (Table 3). While overall race was not associated with other complications (p = 0.06), Black infants with RI greater than or equal to the median had increased odds of complications (OR 1.50, 95% CI 1.08–2.10). These infants also had decreased number of outpatient encounters (OR 0.66, 95% CI 0.49–0.89). After adjustment, associations with both LOS (RR 1.67, 95% CI 1.43–1.95) and number of outpatient encounters (OR 0.65, 95% CI 0.47–0.88) remained statistically significant. Additionally, White infants with greater than or equal to the median RI had a decreased number of outpatient encounters in both unadjusted and adjusted analysis (OR 0.71, 95% CI 0.53–0.94) compared to White infants with less than median RI.

## Discussion

In this study we assessed the association of RI, EI, and race with various clinical outcomes in infants with complex CHD. Degrees of RI and EI were associated with differences in resource utilization. Black race was associated with increased LOS, even when controlling for RI. We found that the infants with both Black race and high levels of RI were at highest risk of poor outcomes, including increased LOS, increased mortality, and decreased outpatient encounters. These data suggest that both RI and EI, as well as race, and their additive effects are important factors in outcomes for infants with complex CHD.

We found that patients with Black race had poorer outcomes than those with White race, even when controlling for RI. This adds to the already vast body of literature that patients from races other than White are at increased risk for poor outcomes, with Black individuals most significantly affected.^29^ These disparities, which have not improved over the last 35 years are likely due to factors at varying levels of the healthcare system including population, systemic, institutional, and individual levels as well as the persistence of systemic racism within our societal structures.^1,30^ Access to and quality of care may be variable based on patient race.^31^ Future interventions designed to address these disparities must target all levels to ensure equitable outcomes.

Differences in outcomes for patients with CHD are likely influenced not only by individual patient characteristics, such as race, but the environments in which they live. We found differences in resource utilization based on RI with patients having decreased outpatient encounters and increased inpatient encounters with increasing isolation at lower levels of RI and decreased inpatient encounters with increasing isolation at higher levels of RI. Decreased outpatient utilization may be due to difficulty in getting to appointments which may be remote from more racially isolated areas with more challenging access to transportation. This may in turn lead to more need for hospitalization due to inability to manage conditions as an outpatient. An unexpected finding was decreased inpatient encounters with increasing isolation at higher levels of RI. This may be due to either not having to present or reluctance to present to the hospital. Further investigation is needed to determine the mechanism of this relationship.

Patients with CHD often require complex care outside of the hospital, including multiple outpatient appointments and admissions and home health equipment requirements such as feeding tubes and oxygen saturation monitoring, placing a large burden on families. These burdens can be exacerbated by neighborhood attributes, including racial segregation. Racial isolation has been shown to cause poor outcomes in type 2 diabetes mellitus,^19^ preterm birth, ^32^ and low birth weight^17^. These differences may be due to concentrated socioeconomic disadvantage and lack of resources in these neighborhoods. Importantly, we found that there is also a cumulative effect of race and RI, with infants in this cohort with Black race and RI greater than the median having highest odds of poor outcomes. This was especially notable with the significantly increased odds of 3.4 of death in this group. This may be due to concentrated levels of poverty, poor housing quality, decreased access to healthy food, and decreased access to transportation. While race, a social construct, is not modifiable, these neighborhood characteristics are modifiable. Interventions can be used to address these such a use of community health workers to engage families, improved quality of housing support, and improving transportation access.^33–35^

In children, maternal education level has been tied to poorer outcomes in many areas of health, including CHD, though there have been mixed results with its effect.^2,4,22–24^ Similar to race, it may be that more than just an individual or parents’ education level that affects outcomes, rather the environmental education level, which in turn affects income, housing, transportation, and more. We found that risk of an ED encounter was increased at the highest levels of EI, suggesting that increased EI plays a role in health utilization in infants with CHD. This emphasizes the importance of providing detailed instruction to caretakers of children with complex medical problems at each encounter as well as assessing support for families in caring for their child. Further investigation is warranted into the relationship between EI and other socioeconomic neighborhood level factors, such as income, housing quality, transportation access, and food insecurity.

There are some limitations to this study. This study occurred in a single state in the southeastern United States. It has been shown that RI and EI have different relationships across time and space.^25^ We found that RI was similar across EI groups. This finding may be unique to the population of study, however similar findings may exist in areas with similar geographic characteristics. These data were collected from 2008–2013 yet we still believe it is highly relevant today and likely reflects the current state of RI/EI in CHD as there is evidence that while CHD mortality has decreased, disparities have persisted and community-wide interventions remain in their infancy.^1^ Additionally, it may be that given the increased disparities seen after the COVID-19 pandemic, disparities may be even more relevant than that during the time period for this study. This study focused on a high risk group of infants, those with CHD requiring intervention in the first year of life, for which there are a significant amount of resources in place for close monitoring, especially those with single ventricle physiology.^36^ Previous studies have shown increased risk in those with less complex CHD and this population may be differentially affected by RI and EI.^4^ Given that these data were collected on infants who underwent a surgical procedure, it does not include those that were diagnosed prenatally and chose to terminate, those choosing comfort care, and those who may not have been surgical candidates. These groups may have differing degrees of racial and educational isolation. This study used data extracted from the electronic health record for patient race, which reflects the patient’s self-reported race, which we acknowledge as a social construct rather than biological descriptor.^37^ Additionally, this data set did not include many other important individual level sociodemographic factors (e.g. family structure and income) which also likely play a role in patient outcomes.

Based on these findings, it is important to consider the social context in which patients exist. When assessing patients, we should be identifying not only the clinical risk factors but assessing the sociodemographic risk factors in all children with CHD so these may be addressed just as vigorously. Continued research is needed into the optimal interventions to address these inequities. Additionally, much of the literature has focused on disparities in the outcome of mortality which was seen in our study, however much remains to be done to evaluate the extent to which disparities exist in other important outcome measures, such as LOS, complications, neurodevelopment, and quality of life. Interventions to address all of these outcomes are just as essential for the overall care of children with CHD.

## Conclusion

In this study we assessed the association of RI, EI, and race with various outcomes in infants with complex CHD and found all three were associated with poorer outcomes. These data suggest both environmental factors like RI and EI, as well as individual factors such as race, are important factors in outcomes for infants with complex CHD. Additionally, there is likely a cumulative effect of these factors with infants with Black race and high levels of RI having the highest odds of poor outcomes. Next steps include investigations into targeted interventions to address these social determinants of health and promote health equity.

## Supplementary Material

Supplement 1

## Figures and Tables

**Table 1:** Characteristics and Outcomes of Overall Cohort

Characteristic	N = 1217
Age (days), median (Q1, Q3)	17 (0, 112)
Female, n (%)	527 (43.3%)
Race, n (%)	
Black	270 (22.2%)
Other	39 (3.2%)
Unknown	209 (17.2%)
White	699 (57.4%)
Hispanic Ethnicity, n (%)	150/1101 (13.6%)
Insurance, n (%)	
Medicaid	418 (34.3%)
Private	293(24.1%)
Self-pay	6 (0.5%)
Other	24 (2.0%)
CHD Severity	
Severe	768 (63.4%)
Shunt	369 (30.4%)
Valve	73 (6%)
Other	2 (0.2%)
Preterm Birth	98 (8.1%)
Racial Isolation Index (RI), mean (SD)	0.22 (0.17)
Educational Isolation Index (EI), mean (SD)	0.74 (0.15)
Outcome	
*Initial Hospitalization*	
Length of Stay (days), Median (Q1, Q3)	16 (7, 36)
Surgical Complications, n (%)	
Major Complications	220 (18.3%)
Any other complications	493 (41.4%)
*1 Year Post Discharge*	
Hospital Readmission, n (%)	218 (18.2%)
Days Alive Outside Hospital, median (Q1, Q3)	362 (351, 365)
Death, n (%)	81 (6.8%)
Resource Utilization	
Number of Outpatient Encounters, mean (SD)	5.6 (8.5)
At least 1 Emergency department encounter	237 (19.8%)
At least 1 Inpatient encounter	654 (54.5%)

Demographic data and CHD classification are from qualifying encounter. CHD classification at the qualifying encounter was missing for 5patients. However, patients were required to have a diagnosis of CHD to be in the cohort.

Length of stay and surgical complications were during patienťs index hospitalization. Hospital readmission, days alive and out of hospital, death, arid resource utilization were 1-year post discharge.

17 patients with missing discharge dates are excluded from outcomes analyses

SD = Standard deviation, RI = racial isolation index, EI = educational isolation index

**Table 2:** Association between Race and Outcomes

Outcome	Unadjusted Odds Ratio (95% CI)	p-value	Adjusted Odds Ratio (95% CI)	p-value
Length of Stay^*1^		< .001		< .001
Black or African American	**1.49 (1.25–1.78)**		**1.63 (1.38–1.91)**	
Other	1.29 (0.91–1.83)		1.15 (0.85–1.55)	
Any Major Surgical Complication		0.14		0.271
Black or African American	1.47 (0.98–2.20)		1.43 (0.91–2.25)	
Other	1.46 (0.67–3.16)		1.32 (0.57–3.04)	
Any other complication		0.03		0.285
Black or African American	**1.49 (1.07–2.08)**		1.28 (0.89–1.85)	
Other	1.71 (0.89–3.27)		1.45 (0.72–2.91)	
Readmission		0.14		0.10
Black or African American	1.02 (0.67–1.56)		1.17 (0.73–1.87)	
Other	0.24 (0.06–1.00)		0.22 (0.05–0.97)	
Death		0.02		--
Black or African American	**2.18 (1.21–3.93)**			
Other	0.47 (0.06–3.56)			
Number of OP Encounters		0.54		0.71
Black or African	0.84 (0.61–1.14)		0.90 (0.64–1.25)	
American				
Other	0.93 (0.51–1.69)		0.83 (0.45–1.53)	
At least 1 ED encounter		0.06		0.15
Black or African American	**1.63 (1.08–2.44)**		1.51 (0.98–2.34)	
Other	0.94 (0.39–2.30)		0.85 (0.34–2.13)	
At least 1 IP Encounter		0.56		0.80
Black of African American	1.06 (0.76–1.49)		0.93 (0.64–1.34)	
Other	1.44 (0.73–2.84)		1.19 (0.58–2.44)	

*1 Length of stay was modeled using negative binomial regression. The estimate presented is the incidence rate ratio. All other outcomes were modeled using logistic regression and the estimate presented is the odds ratio.

All models include the racial isolation index, in addition to race. Adjustment variables include age (days), sex, ethnicity, disease severity, preterm birth and length of index hospitalization (excluded from LOS and surgical complication outcomes).

SD = Standard deviation, RI = racial isolation index, EI = educational isolation index, OP = outpatient, ED = Emergency department, IP = inpatient

**Table 3:** Association Between Race and Racial Isolation and Outcomes

	Unadjusted		Adjusted	
Outcome	Odds Ratio (95% CI)^*2^	p-value	Odds Ratio (95% CI)^*2^	p-value
Index Hospitalization				
Length of Stay		<.001		<.001
White, RI ≥ Median	1.10 (0.93–1.30)		1.03 (0.89–1.20)	
Black, RI < Median	1.29 (0.94–1.77)		**1.48 (1.09–2.01)**	
Black, RI ≥ Median	**1.69 (1.42–2.02)**		**1.67 (1.43–1.95)**	
Surgical Complications				
Any major complication		0.13		0.235
White, RI ≥ Median	0.80 (0.53–1.22)		0.87 (0.55–1.37)	
Black, RI < Median	1.12 (0.54–2.33)		0.98 (0.41–2.37)	
Black, RI ≥ Median	1.39 (0.93–2.07)		1.42 (0.92–2.19)	
Any other complication		0.06		0.181
White, RI ≥ Median	1.02 (0.74–1.40)		1.15 (0.82–1.63)	
Black, RI < Median	1.51 (0.84–2.72)		1.62 (0.82–3.21)	
Black, RI ≥ Median	**1.50 (1.08–2.10)**		1.43 (1.00–2.05)	
1 Year Post Discharge				
Readmission		0.16		--^*1^
White, RI ≥ Median	0.64 (0.43–0.97)			
Black, RI < Median	0.60 (0.26–1.38)			
Black, RI ≥ Median	0.84 (0.56–1.28)			
Death		0.001		--^*1^
White, RI ≥ Median	1.57 (0.79–3.10)			
Black, RI < Median	1.98 (0.64–6.09)			
Black, RI ≥ Median	**3.40 (1.84–6.31)**			
*Resource Utilization*				
Number of OP Encounters		0.02		0.02
White, RI ≥ Median	**0.71 (0.53–0.94)**		**0.69 (0.51–0.92)**	
Black, RI < Median	0.75 (0.44–1.29)		0.97 (0.53–1.79)	
Black, RI ≥ Median	**0.66 (0.49–0.89)**		**0.65 (0.47–0.88)**	
At least 1 ED Encounter		0.10		--^*1^
White, RI ≥ Median	0.83 (0.54–1.27)			
Black, RI < Median	1.35 (0.66–2.75)			
Black, RI ≥ Median	1.45 (0.97–2.16)			
At least 1 IP Encounter		0.009		--^*1^
White, RI ≥ Median	1.30 (0.95–1.77)			
Black, RI < Median	0.71 (0.39–1.28)			
Black, RI ≥ Median	**1.62 (1.16–2.26)**			

Median RII for white and Black patients combined is 0.174.

Length of stay and number of OP encounters were modeled using negative binomial regression. Theestimate presented is the rate ratio. All other outcomes were modeled using logistic regression andthe estimate presented is the odds ratio.

Adjustment variables include age (days), sex, ethnicity, disease severity, preterm birth and length ofindex hospitalization (excluded from LOS and surgical complication outcomes).

*1 There were too few events in one group to run adjustment models for readmission, death and thebinary resource utilization outcomes

*2 Ref group used was White, RII < Median

## Abbreviations

CHD: Congenital Heart Disease
SES: socioeconomic status
RI: Racial isolation
EI: Educational isolation
ED: Emergency department
LOS-: length of stay
NHW: Non Hispanic White
NHB: Non-Hispanic Black
CI: Confidence Interval
SD: standard deviation
OR: odds ratio
