# Supplementary material for: Racial and Educational Isolation are Associated with Worse Outcomes in Congenital Heart Disease
Source: Res Sq. 2024 Oct 30:rs.3.rs-5290293. Preprint. [Version 1] doi: 10.21203/rs.3.rs-5290293/v1 (PMC11581119; doi:10.21203/rs.3.rs-5290293/v1)
Supplement: Supplement 1 [file NIHPPRS5290293V1-supplement-1.pdf]

Supplementary Files

This is a list of supplementary files associated with this preprint. Click to download.

- RIEISuppFigureFinal.docx
- RIEIsupptablesPC.docx
